# Formation of chloroplast protrusions and catalase activity in alpine *Ranunculus glacialis* under elevated temperature and different CO_2_/O_2_ ratios

**DOI:** 10.1007/s00709-015-0778-5

**Published:** 2015-02-21

**Authors:** Othmar Buchner, Tim Moser, Matthias Karadar, Thomas Roach, Ilse Kranner, Andreas Holzinger

**Affiliations:** Institute of Botany, Functional Plant Biology, University of Innsbruck, Sternwartestrasse 15, 6020 Innsbruck, Austria

**Keywords:** Ascorbate peroxidase, Hydrogen peroxide, Photorespiration, Stromules, Ultrastructure

## Abstract

Chloroplast protrusions (CPs) have frequently been observed in plants, but their significance to plant metabolism remains largely unknown. We investigated in the alpine plant *Ranunculus glacialis* L. treated under various CO_2_ concentrations if CP formation is related to photorespiration, specifically focusing on hydrogen peroxide (H_2_O_2_) metabolism. Immediately after exposure to different CO_2_ concentrations, the formation of CPs in leaf mesophyll cells was assessed and correlated to catalase (CAT) and ascorbate peroxidase (APX) activities. Under natural irradiation, the relative proportion of chloroplasts with protrusions (rCP) was highest (58.7 %) after exposure to low CO_2_ (38 ppm) and was lowest (3.0 %) at high CO_2_ (10,000 ppm). The same relationship was found for CAT activity, which decreased from 34.7 nkat mg^−1^ DW under low CO_2_ to 18.4 nkat mg^−1^ DW under high CO_2_, while APX activity did not change significantly. When exposed to natural CO_2_ concentration (380 ppm) in darkness, CP formation was significantly lower (18.2 %) compared to natural solar irradiation (41.3 %). In summary, CP formation and CAT activity are significantly increased under conditions that favour photorespiration, while in darkness or at high CO_2_ concentration under light, CP formation is significantly lower, providing evidence for an association between CPs and photorespiration.

**Electronic supplementary material:**

The online version of this article (doi:10.1007/s00709-015-0778-5) contains supplementary material, which is available to authorized users.

## Introduction

Extensions of different organelles, including protrusions and stromules, are a frequently observed phenomenon (Gray et al. 2001; Hanson and Sattarzadeh 2008, 2011; Mathur et al. 2012), but their physiological functions remain largely unknown. In chloroplasts, different forms of stroma-filled extensions of the plastid envelope were described more than a century ago (e.g. Senn 1908; Heitz 1937; reviewed by Gray et al. 2001), referred to as protuberances and later as proliferations (Lütz and Moser 1977; Lütz 1987), stromules (Köhler and Hanson 2000) or chloroplast protrusions (Buchner et al. 2007a,b; Holzinger et al. 2007a,b). Stromules were described as long and thin stroma-filled tubules (diameter 0.4–0.8 μm, length up to 65 μm; Gray et al. 2001), similar to beak-like chloroplast protrusions (CPs) of the chloroplast envelope (diameter 3–5 μm, length 3–5 μm; Holzinger et al. 2007a) but significantly narrower. Both stromules and CPs may form and withdraw rapidly. For comprehensive literature concerning stromule activity and dynamics, see Köhler and Hanson (2000), Kwok and Hanson (2003, 2004a,b) and Hanson and Sattarzadeh (2008).

Stromules have been suggested to be involved in the protein trafficking (Köhler et al. 1997; Gray et al. 2001), and the mechanisms are currently being investigated (see Hanson and Sattarzadeh 2011, 2013; Schattat et al. 2012, 2015). Stromules differ from CPs in shape, and therefore, possibly in function. Moser et al. (2015) demonstrated that under natural environmental conditions CP formation in leaves of *Ranunculus glacialis* follows a pronounced diurnal rhythm, and that CPs are most abundant in the afternoon and not related to temperature or irradiation stress. However, it has been suggested that CPs may contribute to the adaptation mechanisms of plants in extreme habitats such as in alpine and polar regions with short vegetation periods (Lütz and Engel 2007; Lütz 2010; Lütz et al. 2012).

Formation of CPs was shown to increase after acid mist treatment in Sitka spruce (Wulff et al. 1996) and in salt-stressed *Mesembryanthemum crystallinum* (Paramanova et al. 2004) and rice leaves, the latter of which contained crystalline inclusions within CPs alongside immunolabelled ribulose 1,5-bisphosphate carboxylase/oxigenase (rubisco) (Yamane et al. 2012). In early TEM studies on *R. glacialis* (Lütz 1987) and later in other high alpine and polar plant species (Gielwanowska and Szczuka 2005; Lütz et al. 2006; Holzinger et al. 2007b; Lütz and Engel 2007; Lütz 2010; Lütz et al. 2012), CPs were frequently found to be located in close spatial proximity to mitochondria and peroxisomes, suggesting a link between photorespiration and CP formation. However, quantitative evidence for this link is still missing. During photosynthesis, rubisco catalyses CO_2_ fixation; however, under increasing temperatures, rubisco increasingly reacts with O_2_ (photorespiration) and the resulting oxidation of ribulose-1,5-bisphosphate (RuBP) produces glycolate. This is broken down by glycolate oxidase in peroxisomes producing H_2_O_2_ which is detoxified by catalase (Mhamdi et al. 2012). Moser et al. (2015) found that CP formation was significantly reduced after exposure of *R. glacialis* to 2000 ppm CO_2_ and 2 % O_2_, suggesting that a restriction of photorespiration, as achieved under these conditions, is involved in the withdrawal of CPs.

We used *R. glacialis* as a model alpine species to analyse the suggested link between photorespiration and CP formation in more detail. To achieve this, we measured CP formation in leaves exposed to solar irradiation at varying CO_2_ concentrations to favour or restrict photorespiration, and assessed the activity of catalase (CAT).

## Material and methods

### Plant material and study site

*R. glacialis* is one of the highest ascending (>4000 m a.s.l.) seed plants in the European Alps. As a pioneer species it prefers scree and humid siliceous substrates in the sub-nival and nival zone and is also present in arctic and subarctic regions (Schönswetter et al. 2003). Individuals of *R. glacialis* were carefully excavated near the Timmelsjoch pass (2563 m a.s.l.; Ötztal Alps, Tyrol, 46° 54′ N/11° 09′ E; 18 July 2013), potted and left in their natural habitat for 3 weeks. The potted plants were transported to the ‘Alpine Garden Patscherkofel’ near Innsbruck (1950 m a.s.l.). For acclimation, the plants were partially shaded and carefully watered for 1 week until the experiments started on 15 August 2013.

### Exposure to different CO_2_ concentrations

**Fig. 1:**
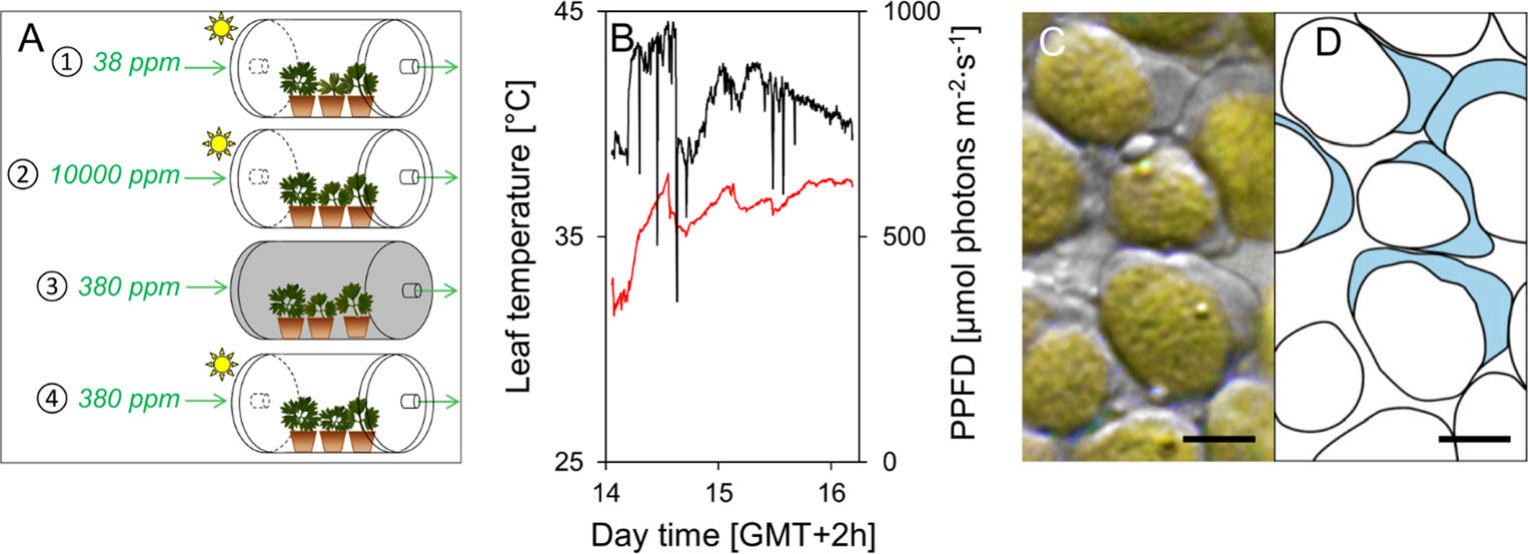
Experimental set-up for determining the effect of different CO_2_ concentrations on the formation of chloroplast protrusions (CPs) in leaves of *R. glacialis*. On 15 August 2013, **a** potted plants (*n* = 5) were placed inside cylindrical exposure chambers made of highly transparent Plexiglas and exposed to natural solar irradiation with the exception of (3) which was kept in darkness. During the 2.5 h exposure, air with different CO_2_ concentrations was streamed through the chambers. *1* 10,000 ppm, *2* 38 ppm, while *3* and *4* had normal CO_2_ concentration (380 ppm). **b** Mean leaf temperature (*red line*; *n* = 16) and photosynthetically active photon flux density (PPFD; *black line*) for the whole duration of the exposure. **c** Typical CPs in leaf mesophyll of *R. glacialis* immediately after the 2.5 h exposure at 38 ppm CO_2_ and at natural solar irradiation. The DIC microscopy image clearly shows CPs as broad and beak-like, stroma-filled extensions of the chloroplast envelope. **d** Schematic drawing of **c**; *light blue* areas indicate CPs. *Horizontal bars* 2 μm

To determine the impact of different CO_2_ concentrations on CP formation under natural solar irradiation or darkness, four experimental conditions (ECs) were applied for 2.5 h. The CO_2_ concentrations in EC1 and EC2 were controlled to stimulate and prevent photorespiration, respectively. EC1 and EC2 comprised CO_2_ concentrations of 38 and 10,000 ppm, respectively, under natural solar irradiation. EC3 and EC4 comprised atmospheric CO_2_ concentrations (380 ppm) either kept in the dark using metal cylinders or under natural solar irradiation, respectively (Fig. 1a). Environmental conditions were maintained using highly transparent Plexiglas cylinders (200 × 350 mm, XT 29070, Röhm, Darmstadt, Germany; spectral transmittance: see Suppl. 1). Each cylinder contained five individuals of *R. glacialis* that were provided with variable CO_2_ concentrations (Airliquide, Schwechat, Austria) at a constant flow rate of 4000 ml min^−1^. Leaf temperatures of four individual leaves in EC1, EC2 and EC4 were monitored every 5 s by software-controlled heat tolerance testing system (HTTS; Buchner et al. 2013) to enable regulating EC3 to the same temperature of EC1, EC2 and EC4.

### Sampling and preservation

At the end of a 2.5-h exposure, the chambers were quickly opened and leaf samples were taken (one per individual) and cut into 2 × 2 mm pieces that were fixed in 2.5 % glutaraldehyde (GA) in sodium cacodylate buffer (50 mM, pH 7.0). After 1.5 h of immersion in the fixative, leaf pieces were rinsed with and subsequently stored in the same buffer at 5 °C in darkness. For determining enzymatic activities, the remaining leaves were frozen in liquid nitrogen (LN_2_), intermediately stored at −80 °C and lyophilized (Lyovac GT 2, Leybold-Heraeus, Köln, Germany) for 5 days. Prior to chemical analysis, dry samples were ground (Tissue Lyser II, Qiagen, Venlo, the Netherlands) at a speed of 30 Hz for 2 × 45 s and cooled with LN_2_.

### Numeric assessment of chloroplast protrusions

Semi-thin sections (30 μm) were sliced from the GA-fixed leaf samples and analysed using an inverted microscope with differential interference contrast (DIC) optics (Axiovert 200 M; Plan-Apochromat 63 × 1.4 NA; Carl Zeiss, Jena, Germany). According to the method of Moser et al. (2015), the palisade parenchyma was photographed (Axiocam MCR 5, Carl Zeiss, Jena, Germany) and for each sample 10 individual cells were randomly selected. Stacks of images showing the same cell were analysed at different focal planes (Adobe Photoshop CS2, Adobe Systems Inc., San José, CA, USA). For each cell, 10 chloroplasts were thoroughly screened for CPs. Only chloroplasts positioned slightly off the cell wall, and not concealed by cell-wall fragments or other structures, were selected for further investigation. The relative proportion of chloroplasts with CPs (rCP) was calculated for each cell screened (1).

1 $$ rCP\%=100\%\cdot \frac{n(CP)}{n} $$

*n*(CP): number of chloroplasts showing at least one CP
*n*: number of chloroplasts inspected

### Determination of enzyme activities

#### Sample preparation

Twenty milligrams of lyophilized and ground leafs were extracted in 1 ml 50 mM Sørensen’s buffer, pH 7, with 1 mM EDTA and vortexed for 15 s. The suspension was centrifuged at 4 °C for 5 min at 12,000 *g* and 600 μl of the supernatant was diluted with 1400 μl of extraction buffer. Enzymes were purified from low molecular weight compounds that interfered with enzyme assays with PD10 Sephadex® G-25 desalting columns (GE Healthcare, Chalfont St Giles, UK) with centrifugation (4 °C, 1000 *g*, 2 min). The resulting extract was kept on ice prior to measurements.

#### Catalase (CAT; 1.11.1.6) and ascorbate peroxidase (APX; EC 1.1.11.1) activities

CAT activity was measured by combining 100 μl of the extract with 620 μl of extraction buffer and 80 μl of 150 mM H_2_O_2_. The breakdown of H_2_O_2_ was measured by following the absorbance decrease at 240 nm (*ε* = 43.6 M^−1^ cm^−1^) for 2 min. APX activity was measured by combining 150 μl of extract with 820 μl extraction buffer, 20 μl of 10 mM ascorbate solution and 15 μl of 15 mM H_2_O_2_. The breakdown of ascorbate was measured by following the absorbance decrease at 265 nm (*ε* = 7.0 mM^−1^ cm^−1^) for 2 min. For CAT and APX activities, three technical replicates were measured for each biological replicate (*n* = 5), and activity was normalized to dry mass.

### Statistics

Correlation analysis and one-way ANOVA followed by related post hoc tests (Duncan, Games-Howell) to determine significant differences between means were calculated by statistical software (SPSS 21, IBM, Armonk, NY, USA).

## Results

### Leaf temperature and irradiation during the exposure phase

During the treatment, the photosynthetically active photon flux density (PPFD) varied from 360 to 967 μmol photons m^−2^ s^−1^ (mean 715), which is below the maxima that may occur in the field (>2500 μmol photons m^−2^ s^−1^) but in the range of mean PPFD during daytime (Buchner, unpublished data). Leaf temperatures of the four different ECs were around 36 °C and almost identical (Table 1) and never fell below 31 °C (Fig. 1b), whereas short leaf temperature maxima up to 41.9 °C occurred. At natural growing sites of *R. glacialis* mean leaf temperatures during daytime are typically lower, but maximum half hourly mean values around 37–38 °C occur occasionally (Buchner et al. 2015; Moser et al. 2015) and do not cause any leaf damage (Larcher et al. 1997; Buchner et al. 2015). Even short exposure to 41.9 °C as applied here does not induce lethal leaf damage (Buchner et al. 2015), but it stimulates photorespiration because the specificity of rubisco to CO_2_ over O_2_ is reduced as is the solubility of CO_2_ (Brooks and Farquhar 1985).

**Table 1:** Impact of different CO_2_/O_2_ ratios under elevated temperature on the formation of CPs and CAT and APX activities

Experimental condition [EC]	CO_2_ [ppm]	PPFD [μmol photons m^−2^ s^−1^]	TL [°C]	rCP [%]	CAT [nkat mg^−1^ DW]	APX [nkat mg^−1^ DW]
EC 1	38	715	35.9/39.2	58.7	34.7	0.34
EC 2	10,000	715	35.9/39.8	3.0	18.4	0.27
EC 3	380	0	36.3/41.9	18.2	20.4	0.29
EC 4	380	715	36.6/41.0	41.3	22.2	0.31

Individual *R. glacialis* plants were exposed to different experimental conditions [EC]. During exposure, CO_2_ concentration and leaf temperature (TL; mean/maximum) were controlled, and mean solar irradiation (PPFD) was monitored. After 2.5 h of exposure, the relative frequency of chloroplast protrusions (rCP) and the activities of catalase (CAT) and ascorbate peroxidase (APX) were determined

### Impact of CO_2_ concentration on the formation of CPs

**Fig. 2:**
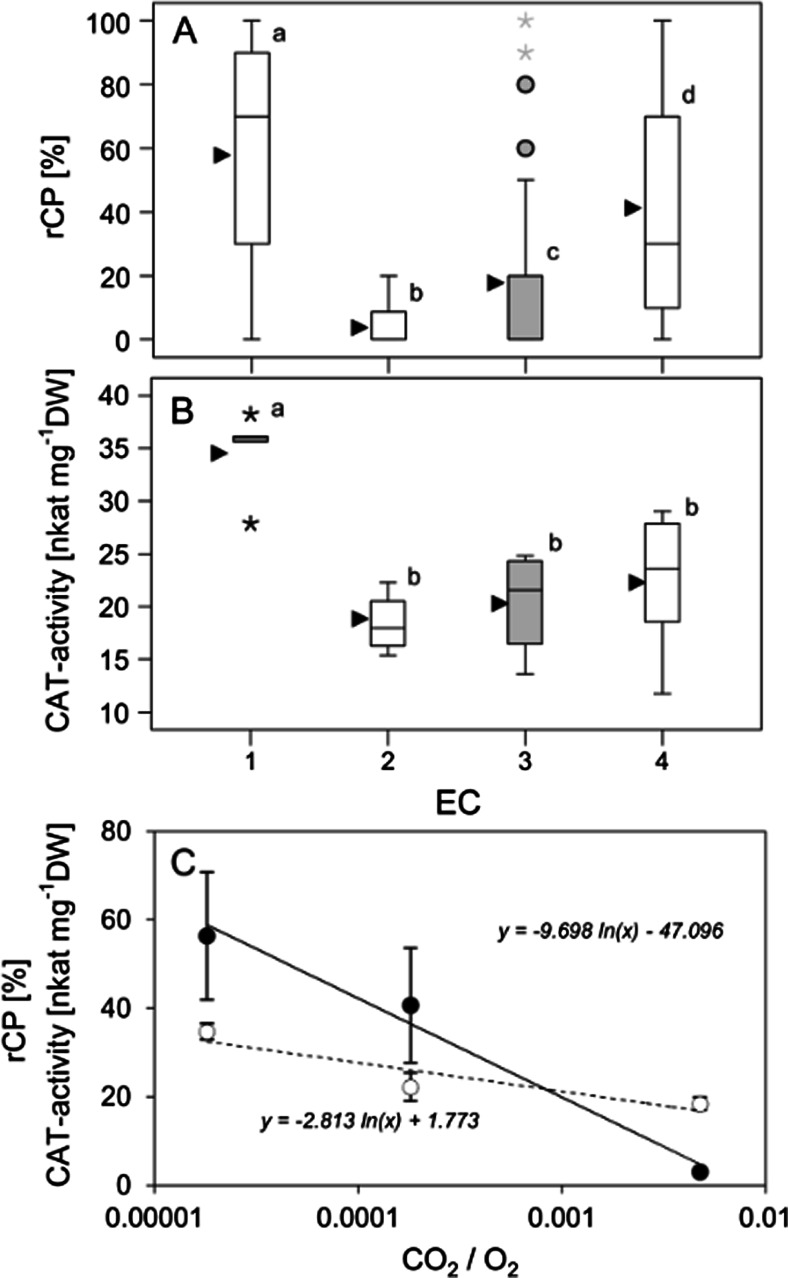
Effect of a 2.5-h exposure of *R. glacialis* individuals at different CO_2_ concentrations on the formation of CPs and on catalase activity. Depending on the experimental conditions (ECs) the following CO_2_ concentrations were applied: *1* 38 ppm, 2 10,000 ppm, and *3* and *4* 380 ppm. *1*, *2* and *4* where exposed to natural solar irradiation (mean PPFD = 715 μmol photons m^−2^ s^−1^), while *3* was kept in darkness (*grey boxes*). **a** Relative proportion of chloroplasts with at least one chloroplast protrusion (rCP); **b** catalase activity in response to the different treatments; **c** rCP (*black circles*) of the light-exposed samples significantly correlates with the CO_2_/O_2_ ratio in accordance with a logarithmic function (*solid line*). This is also true for CAT activity (*white circles*, *dashed line*). *Box plots*: *horizontal lines* indicate the median and the 25th and the 75th percentiles; *whiskers* extend to 1.5 times box-height; *circles* represent outliers, *asterisks* indicate extreme outliers that have values more than three times box-height; *black triangles* indicate arithmetic means. Significant differences (*P* < 0.05) between groups are indicated by different characters (one-way ANOVA followed by Duncan’s (**a**) and Games-Howell (**b**) post hoc tests)

In DIC images, CPs were easily identifiable as broad, stroma-filled lobes (Fig. 1c, d). Although CPs were present in all ECs, they were most abundant (mean ± SE) after exposure to light under 38 ppm CO_2_ (58.7 % ± 4.6). In contrast, rCP was lowest after exposure to light under 10,000 ppm CO_2_ (3.0 % ± 0.7). Exposure to solar irradiation at the natural ambient CO_2_ concentration (380 ppm) led to an rCP of 41.3 % ± 4.4, while the same treatment in the dark led to an rCP of 18.2 % ± 4.2. Mean values of rCP differed significantly (*P* < 0.05) between all ECs (Fig. 2a).

### Impact of CO_2_ concentration on CAT and APX activities

CAT activity was significantly higher (*P* < 0.05) after light exposure under 38 ppm CO_2_ (34.7 nkat mg^−1^ DW ± 1.8) compared to light exposure under 10,000 ppm CO_2_ (18.4 nkat mg^−1^ DW ± 1.5). Exposure to natural CO_2_ concentrations (380 ppm) resulted in an enzyme activity of 22.2 nkat mg^−1^ DW ± 3.2 in the light and 20.4 nkat mg^−1^ DW ± 2.6 in darkness (Fig. 2b). Results of EC2, EC3 and EC4 did not differ significantly from each other (*P* > 0.05). No significant differences were found for APX activity between the four ECs. An overview of rCP, CAT and APX activities subsequent to the exposure to the different ECs is given in Table 1.

### rCP and CAT activities in relation to CO_2_/O_2_ ratio

The CO_2_/O_2_ ratio significantly affected CP formation and CAT activity. In the light (EC1, EC2, EC4), rCP and CAT activities significantly (*P* = 0.001) correlated negatively with the CO_2_/O_2_ ratio (Spearman’s rho = −0.756 and −0.771, respectively) (Fig. 2c). Furthermore, rCP and CAT activities were positively correlated (Spearman’s rho = 0.666, *P* = 0.011). However, no correlations were found between APX activity and the CO_2_/O_2_ ratio or rCP (data not shown).

## Discussion

### Photorespiration and CP formation

It is believed that photorespiration requires close spatial proximity of chloroplasts, peroxisomes and mitochondria to allow transport of metabolites between these organelles (Douce and Neuburger 1999; Eisenhut et al. 2013). It has been suggested that the formation of CPs supports photorespiration by bridging gaps between organelles (Lütz et al. 2012; Hanson and Sattarzadeh 2011) and by enlarging the chloroplast surface to facilitate envelope-bound transport (Lütz 1987; Lütz 2010; Holzinger et al. 2007b; Lütz and Engel 2007). Furthermore, Sage and Sage (2009) suggested that CPs (or stromules) may also operate as a photorespiratory CO_2_-scavenging system that supports re-fixation of photorespiratory-released CO_2_. Catalase, which is essential in scavenging H_2_O_2_ produced from photorespiration, is almost exclusively located in peroxisomes and also plays a role in stress response (Feierabend 2005; Wingler et al. 2000; Mhamdi et al. 2012). Accumulation of H_2_O_2_ was shown to occur in microbodies of *R. glacialis* using diaminobenzidine (Lütz 1987), which is a stain commonly used for H_2_O_2_ in relation to peroxidase activity (e.g. Roach et al. 2010). However, it was not known if CAT activity was related to CP formation.

Hydrogen peroxide can be scavenged by several enzymes, including CAT and peroxidases. APX plays a key role in the ascorbate-glutathione cycle, which serves to scavenge H_2_O_2_ (Foyer and Noctor 2011). However, only CAT activity but not APX activity correlated with CP formation, which suggests that there was a need for enhanced H_2_O_2_ scavenging in peroxisomes rather than chloroplasts. Interestingly, this indicates that the low CO_2_ treatment used to promote photorespiration apparently did not induce the Mehler reaction, agreeing with a recent rethinking that the Mehler reaction is restricted under low CO_2_ conditions (Noctor et al. 2014; Roach et al. 2015). The exposure to varying CO_2_/O_2_ ratios allowed us to modulate photorespiration, showing that CP formation positively correlates with CAT activity (Fig. 2a, b), supporting the hypothesis that CP formation and photorespiration are linked, although a causal relationship is still to be confirmed. Furthermore, it will be interesting to study if organelle extensions such as stromules and CPs also support signalling pathways (Noctor et al. 2007), such as retrograde signalling, which is essential for coordinating cellular activities during plant stress response (Kwok and Hanson 2004a; Fernández and Strand 2008).

### Chloroplast protrusion—a multifaceted phenomenon

Chloroplast protrusions are not solely formed during photorespiration, but also seem to have other roles. We show that CPs were also formed under conditions that do not induce photorespiration (Fig. 2a). If the only role of CPs was in photorespiration, no CPs would be formed in the dark. However, rCP was not zero after exposure to 380 ppm CO_2_ in darkness. In *R. glacialis*, Moser et al. (2015) observed highest rCP values at moderately solar irradiation and moderately elevated leaf temperatures with a significant correlation between leaf temperature and rCP. Furthermore, rCP at 50 ppm did not differ significantly from that at 370 ppm CO_2_, indicating that photorespiration was not the main reason for CP formation, because leaf temperature was only 25 °C (compared to ~36 °C used here). In *Arabidopsis*, CP formation increased with temperature, likely supporting the increased transport of metabolites required for increased metabolic rates at high temperature (Holzinger et al. 2007a). Even in darkness CP formation apparently may support metabolite transport out of the chloroplast during the degradation of transitory starch (Schleucher et al. 1998).

Recent results indicate that increased CP formation could also be related to stress factors. Our results and those of Moser et al. (2015) do not strongly indicate that CPs are formed in response to temperature and irradiation stress. On the other hand, in rice (Yamane et al. 2012) and soybean (He et al. 2014), high salt concentration promoted the formation of CPs and rubisco-containing bodies. In wheat seedlings, protrusions of the chloroplast envelope were shown to be increased during water stress (Freeman and Duysen 1975). Chloroplast swelling and the occurrence of large thylakoid-free areas have also been described in context with chilling or sublethal freezing (Ciamporová and Trginová 1999; Stefanowska et al. 2002) or after heat stress (Larcher et al. 1997). Furthermore, Ishida et al. (2014) showed that vesicles originating from stromules or CPs may be involved in autophagic processes in context with nutrient recycling and chloroplast function maintenance.

In summary, this short communication shows a strong correlation between CP formation and CAT activity, in support of the hypothesis that photorespiration is linked with CP formation, and that CP formation is a multifaceted phenomenon with more than one physiological role.

## Electronic supplementary material

Below is the link to the electronic supplementary material.

ESM 1 (PDF 28 kb)
