# Supplementary material for: Formation of chloroplast protrusions and catalase activity in alpine Ranunculus glacialis under elevated temperature and different CO2/O2 ratios
Source: Protoplasma. 2015 Feb 21;252(6):1613–9. doi: 10.1007/s00709-015-0778-5 (PMC4628086; doi:10.1007/s00709-015-0778-5)

Suppl. 1 Spectral transmittance (Ψ) of Plexiglas® XT Clear 29070 (thickness: 3 mm) in the ultraviolet and visible range (after data from Röhm, Darmstadt, Germany)

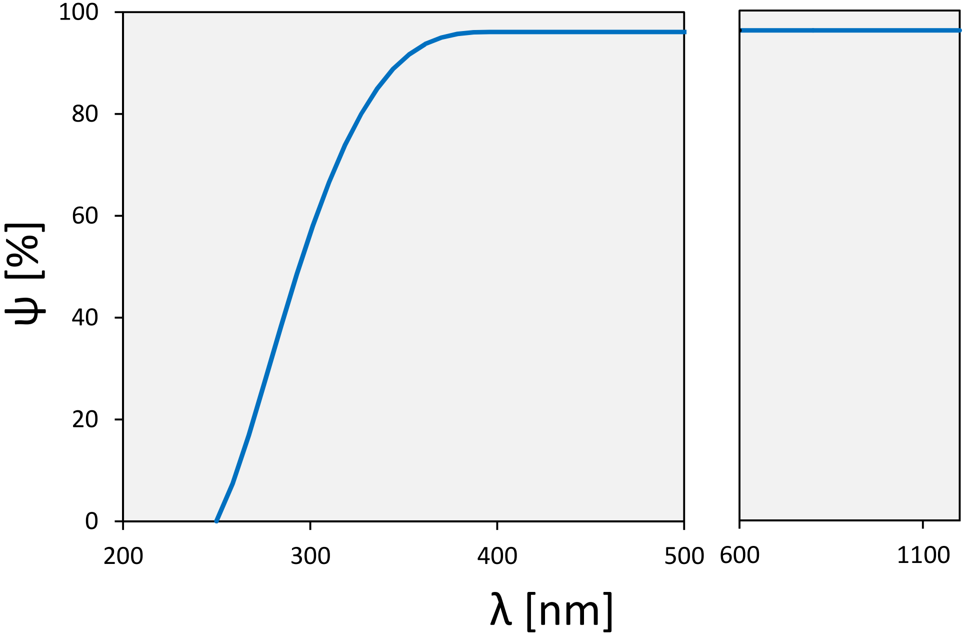

Supplement: Supplementary file 1 — (PDF 28 kb) [file 709_2015_778_MOESM1_ESM.pdf]
